# Research Progress of Liquid Biopsy Based on DNA Methylation in Tumor Diagnosis and Treatment

**DOI:** 10.3390/biom14121634

**Published:** 2024-12-19

**Authors:** Yunxia Bao, Xianzhao Wang, Bingjie Zeng, Yichun Shi, Yiman Huang, Yiwen Huang, Shuang Shang, Liang Shan, Lifang Ma

**Affiliations:** 1 Department of Clinical Laboratory Medicine, Shanghai Chest Hospital, Shanghai Jiao Tong University School of Medicine, Shanghai 200030, China; 2 College of Health Science and Technology, Shanghai Jiao Tong University School of Medicine, Shanghai 200025, China; 3 Shanghai Institute of Thoracic Oncology, Shanghai Chest Hospital, Shanghai Jiao Tong University School of Medicine, Shanghai 200030, China

**Keywords:** DNA methylation, tumor, epigenetic, liquid biopsy

## Abstract

Liquid biopsy has been gradually applied to the clinical diagnosis and treatment of tumors because of its non-invasive and real-time reflection of the tumor status, as well as the convenience of sample collection, which allows the detection of primary or metastatic malignant tumors and reflects the heterogeneity of the tumors. DNA methylation, which is a type of epigenetic modification, is essential in the progression of tumors. This review introduces the common DNA methylation analysis methods and discusses their advantages and disadvantages, focusing on the new progress of DNA methylation-based liquid biopsy in tumor diagnosis and treatment.

## 1. Introduction

Cancer is the most prevalent cause of death in the world. In 2022 the global incidence of new malignant tumors was approximately 20 million cases, and death occurred in approximately 9.7 million cases [1]. According to statistics from the National Cancer Centre of China, it is anticipated that in 2024, there will be roughly 4.8 million new cases of cancer identified within the nation, with an estimated death toll of about 3.2 million linked to these cancers. Both the global and national rates of occurrence and death associated with malignant tumors have been on an upward trend, and it is projected that the number of newly diagnosed cancer cases will surpass 35 million by the year 2050. Therefore, the development of biomarkers related to the early detection, diagnosis, and monitoring of cancer has become an important focus for clinicians with regard to the high incidence and lethality of cancer [2]. However, the development of non-invasive methods for early tumor detection remains a challenge. Liquid biopsy has received widespread attention from clinicians in recent years because of its broad application prospects. Compared with traditional tissue biopsy, liquid biopsy is non-invasive, and sampling is convenient, easy, and can be repeated several times.

Liquid biopsy is a technique that utilizes circulating tumor cells (CTCs), circulating tumor DNA (ctDNA), circulating cell-free DNA (cfDNA), exosomes, and other substances in human blood, urine, and peritoneal fluid for tumor diagnosis, treatment monitoring, and prognosis assessment. It is mainly used to detect biomarkers of tumor origin in patients, so as to effectively judge and provide feedback on the condition, efficacy, and prognosis of tumor patients [3]. Currently, the body fluids applied to liquid biopsy technology mainly include blood, urine, cerebrospinal fluid, sputum, and digestive fluids, with blood testing being the most widely used as testing of other body fluids will have a certain organ specificity. Even though liquid biopsy has been shown to offer great potential for early cancer screening and diagnosis, there are still a few major issues that need to be resolved. The detection method should be extremely sensitive for the early stages of cancer, when there is minimal tumor DNA in bodily fluids and the genomic profile of the initial tumor is unknown [4]. DNA methylation, which is more signal-specific and universal, is gradually coming into the scope of researchers as they delve deeper into their studies.

DNA methylation represents a type of epigenetic modification that has received deep attention from researchers and refers to the process by which DNA methyltransferases add methyl groups to the 5′ position of cytosine in the CpG sequence of DNA to produce 5-methylcytosine [5]. It is capable of regulating gene expression and thus altering genetic expression by causing changes in DNA conformation, DNA stability, and DNA–protein interactions while keeping the DNA sequence unchanged. This chemical modification was identified in mammals before DNA was recognized as genetic material, but it was not until the 1980s that DNA methylation was shown to have a role in cell differentiation and gene control [6]. As researchers delve deeper, DNA methylation has been found to regulate the aging process in the human body. Steve Horvath [7] analyzed more than 5000 samples and found that Hispanics and North American Indians have a significantly slower rate of intrinsic epigenetic aging than Caucasians, revealing that there is significant variability in the degree of DNA methylation between races and that DNA methylation plays a significant role in the development of disease. However, adequate research on methylation diversity and its distribution patterns in natural populations is lacking. A study [8] constructed peripheral blood DNA methylation profiles and regulatory models for the Uyghur and Han Chinese populations in Xinjiang, revealing greater variability in the methylation of the SEPT8 gene between the two groups.

An increasing amount of research suggests that methylation occupies a crucial role in tumorigenesis and progression, and irregular patterns of DNA methylation are strongly linked to various human diseases, including cancer [9]. In contrast to their normal counterparts, numerous cancer cells are characterized by abnormal methylation patterns, with hypermethylation and hypomethylation regulating gene expression to varying degrees [10]. DNA methylation typically happens early in tumor development and is thought to be specific to cancer [11,12]. A great deal of research has shown that tumor target gene methylation assays have good prospects for application and development in early cancer screening, efficacy detection, and prognosis monitoring. In this review, we describe the currently commonly used DNA methylation detection methods and discuss the new research progress of DNA methylation-based liquid biopsy in tumor diagnosis and treatment, as well as the challenges it faces.

## 2. DNA Methylation Detection Methods

**Figure 1:**
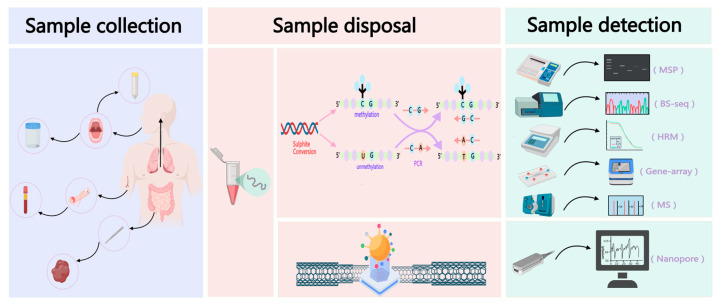
Common DNA methylation analysis workflow, including sample collection, sample disposal, and several common methods of sample detection.

As DNA methylation has gradually become a hot topic for researchers, multiple methods have been exploited to detect DNA methylation. Each method for DNA methylation has its own characteristics and application scenarios, which can be markedly divided into two main types: bisulfite conversion-based methods and non-bisulfite conversion methods. Table 1 outlines the advantages and disadvantages of the major detection methods that are currently in use. In this paper, we focus on several commonly used bisulfite conversion-based assays (Figure 1). The majority of existing DNA methylation assays rely on bisulfite conversion. The principle of bisulfite conversion is that the unmethylated cytosine (C) is deamidated to uracil (U) after DNA is treated with bisulfite, and at the same time, the methylated cytosine (5-mC) does not change in the process of the conversion. The differences in the methylation modifications are converted into differences in the sequences [13,14], finally identifying whether cytosine is methylated or not by recognizing C or T.

Bisulfite sequencing (BS-seq) was first proposed in 1992 [15]. It is considered the “gold standard” of DNA methylation detection. BS-seq includes several techniques for high-resolution methylation analysis. Bisulfite sequencing PCR(BSP) is currently the most direct and reliable technique to reflect the methylation status of genomes [16], which involves designing primers on sequences flanking CpG islands but not containing CpG sites, simultaneously amplifying and sequencing non-methylated and methylated sequences. The technology exhibits high precision by enabling the determination of methylation status for all CpG islands within the amplified region, but it is time-consuming, cumbersome, and costly [17]. Pyrosequencing [18] is a technology that utilizes an enzyme coupling reaction to accomplish real-time monitoring of bio-optical signals released during DNA synthesis reactions and is capable of sequencing sulfite-treated DNA to derive the methylation level of the corresponding site. This technology can rapidly detect methylation levels and perform qualitative and quantitative analyses of specific sites, but its sequencing length is short and long fragments cannot be analyzed. Whole-genome bisulfite sequencing (WGBS) is based on sulfite-treated DNA combined with high-throughput sequencing technology, which allows genome-wide DNA methylation mapping with single-base resolution. The technology is highly sophisticated and capable of obtaining genome-wide methylation information, but it is costly and not suitable for large numbers of samples [19]. Reduced representation bisulfite sequencing (RRBS) [20] was proposed in 2005, and its main principle is to use restriction endonucleases to enzymatically cleave genomic DNA and electrophorese and recover the desired fragments before bisulfite sequencing. This technique is more precise, less costly, and has high data utilization, but is less reproducible and does not cover complete genome-wide methylation information [21].

Methylation-specific PCR (MSP): Methylation-specific PCR is employed for the analysis of DNA methylation patterns within CpG islands, where DNA is treated with sulfite and a pair of primers are designed for methylated or unmethylated sequences and then amplified by PCR followed by further gel electrophoresis. Primer design is the key to this method, which can be employed to assess the methylation status of specific genes. Even a small amount of DNA can still be detected and analyzed. In the beginning, detection was only achieved by electrophoresis, so it was only possible to qualitatively detect known sequences and not quantitatively to the methylation level, which was easily affected by the amplification efficiency.

Afterward, researchers combined MSP and fluorescence quantitative PCR technology to establish the fluorescence quantification method (MethyLight) by designing TaqMan probes on the basis of the MSP method to achieve the quantitative analysis of DNA methylation levels [22]. This method is easy to operate and does not need further gel electrophoresis; however, a calibration curve needs to be established, which is cumbersome to operate.

Methylation-sensitive high-resolution melting analysis (MS-HRM) [23] is a method for determining methylation levels based on the difference in thermal stability between methylated and non-methylated amplification products after bisulfite treatment. DNA can be amplified using conventional primers. Because the methylated and non-methylated amplification products contain different pyrimidines, the melting curve positions and melting temperatures (T_M_) of the two products change accordingly. By comparing them with the standard methylation level, their overall methylation level can be quickly inferred. However, this technique can only detect the overall methylation level of DNA fragments and cannot detect DNA methylation at specific sites.

Microchip technology: The detection principle is based on the hybridization signals of DNA sequences after sulfite treatment, which are captured by designing specific probes and then analyzed using microarray hybridization, which can simultaneously analyze the methylation status of several genes or the whole genome. Nowadays, mainstream DNA methylation microarrays, including the Infinium HumanMethylation450 BeadChip (450 K), Infinium MethylationEPIC BeadChip (850 K), and 935 K [24], have been added on the basis of 850 K, which can identify the methylation status of multiple samples at the same time and is suitable for the parallel analysis of multiple samples. However, its microchip design has a targeting limitation. The designs of different microchips lead to differences in probe coverage and resolution. The report pointed out that the 850 K chip is based on the 450 K, covering more than 90% of the original CpG, as well as additional ENCODE and FANTOM5 project identification of the enhanced sub-area, and the resolution is greatly enhanced [25].

Mass spectrometry: Mass spectrometry-based DNA methylation is a convenient and fast way to directly detect DNA methylation. Usually, mass spectrometry used for methylation modification analysis can be divided into two methods. A common method is a mass array, the technical principle of which is through base-specific enzymatic reactions and sensitive and reliable mass spectrometry technology. Firstly, it operates after sulfite treatment and then uses T7-promoter-tagged primers for PCR amplification [26]. After a series of enzymatic reactions, it shows the base difference at the methylation site, which can be detected by time-flight mass spectrometry. After a series of enzymatic reactions, the methylation sites show base differences, and the methylation level can be detected by time-flight mass spectrometry, which is suitable for the precise determination of the methylation level of specific DNA fragments. This method has high sensitivity, good specificity, easy data, and visual data; however, the instrument is expensive and operation requires high technical requirements.

Although PCR or sequencing techniques based on sulfite treatment are still the mainstay of DNA methylation analysis, there are still shortcomings, including low conversion efficiency, which limits the analysis of small numbers of samples, and a cumbersome treatment process [27]. More and more researchers are focusing on the newly developed nanopore sequencing technology. Nanopore sequencing technology is a sequencing technology based on single-molecule detection. The principle is that when a single strand of DNA passes through a nanopore protein under the action of electrophoresis, the different bases lead to different changes in the current, and the bases are identified by analyzing the characteristics of the current changes [28]. In 2014, the first commercialized nanopore sequencer (Minl0N) was launched by Oxford Nanopore Technologies, and as the technology continues to be optimized, the precision of base detection has been improved even more, with lower cost consumption [29]. It was found that compared to the traditional bisulfite method of analyzing DNA methylation near transcription start sites, the results of nanopore sequencing could detect hypomethylation on TSS more sensitively, suggesting that nanopore sequencing is more accurate [30]. Nanopore sequencing technology has a wide range of applications in clinical research due to its advantages of being high throughput, with ultra-long sequencing read lengths, simple and portable equipment, and the ability to directly detect base methylation modifications. However, the high sequencing error rate directly affects the results [31]. Therefore, nanopore sequencing technology needs to be continuously optimized to improve the accuracy of sequencing, and it is believed that it may play an increasingly important role in clinical diagnosis and treatment in the future.

**Table 1:** Advantages and disadvantages of several common DNA methylation detection methods.

Skill	Advantages	Disadvantages	References
BSP	Reliable results High precision Detects methylation status of individual CpG sites	High sample requirements and costs Complex and Time-consuming for experiments	[16,17]
Pyrosequencing	Quantifiable analyses High resolution	Reads short fragments	[18]
WGBS	High sensitivity Genome-wide coverage	Cumbersome operation High costs Time-consuming for experiments	[19]
RRBS	High-throughput Detects methylation status of individual CpG sites High data utilization	Low repeatability High sample requirements	[20]
MSP	High sensitivity High specificity Simple operation and wide range of specimen types	Requires primer design Only provides overall methylation information High sample requirements	[22]
MS-HRM	Rapid testing	Only detects the overall methylation level of DNA fragments	[23]
Mass Spectrometry	High sensitivity High specificity Monitors the dynamics of DNA methylation Monitors many types of DNA methylation	High cost Time-consuming Limited accuracy and precision	[26]
Microarray technology	Genome-wide coverage High repeatability A large number of data from public databases	Unfavorable methylation segment analysis	[24]
Nanopore sequencing	Real-time sequencing Reads segment lengths	High sequencing error rate	[27,28,29,30,31]

## 3. Application of Liquid Biopsy Based on DNA Methylation Technology in Tumors

The dynamic regulation of DNA methylation, recognized as a vital epigenetic mechanism in the progression, persistence, and dissemination of cancer, shows potential for use as a therapeutic target and as an indicator for prognosis and diagnosis. Liquid biopsy techniques for DNA methylation have already been applied in clinical settings, and we introduce the various genes currently associated with DNA methylation and tumor research.

### 3.1. DNA Methylation and Head Tumors

Oropharyngeal squamous cell carcinoma (OPSCC) is a tumor of the oropharynx, tonsils, and base of the tongue, The majority of cases, ranging from 60% to 80%, are linked to human papillomavirus 16 (HPV16) [32], and it usually manifests as a neck mass on one side and is often accompanied by lymph node metastasis, with poor survival and prognosis for overall OPSCC patients. Patients with HPV-associated OPSCC are younger, usually non-smoking men [33]. One study showed that 73% of HPV-associated OPSCCs were locally progressive at the time of first diagnosis [34]. Therefore, there is an urgent clinical need to find ways to enable early diagnosis so that treatment can be initiated as early as possible. The suggestion has been made that HPV-associated OPSCC harbors elevated levels of aberrantly methylated DNA in specific genes involved in the cellular life cycle [35]. The methylation status of nine genes (GHSR, ITGA4, RXRG, UTF1, CDH8, FAN19A4, CTNNA2, NEFH, and CASR) was quantitatively confirmed in 70 cases of pharyngeal squamous cell carcinoma using pyrosequencing. Hypermethylation of these nine genes was significantly associated with HPV-1 positivity. Nakagawa [36] found that there are high numbers of DNA methylation isoforms in HPV-associated OPSCC, which were analyzed in 170 OPSCC microarrays by Infinium 450K. Microarray analysis of 170 OPSCC samples, as well as unsupervised hierarchical cluster analysis, showed that among the HPV-positive OPSCC cases with good prognoses, the best prognoses were found in HPV-positive OPSCCs with hypermethylated phenotypes. This shows that the presence of high DNA methylation subtypes exhibited a significant correlation with the prognostic outcome. Kurokawa [37] examined 40 cases of DNA methylation in specimens from OPSCC patients who underwent radiotherapy, and determined the methylation levels of eight marker genes using bisulfite pyrophosphate sequencing, including ROBO1, ULK4P3, MYOD1, LBX1, CACNA1A, IRX4, DPYSL3, and ELAVL2, which revealed that there was a remarkable association between DNA hypermethylation and prognostic outcomes. DNA methylation could be useful as an indicator to monitor the efficacy of OPSCC.

**Figure 2:**
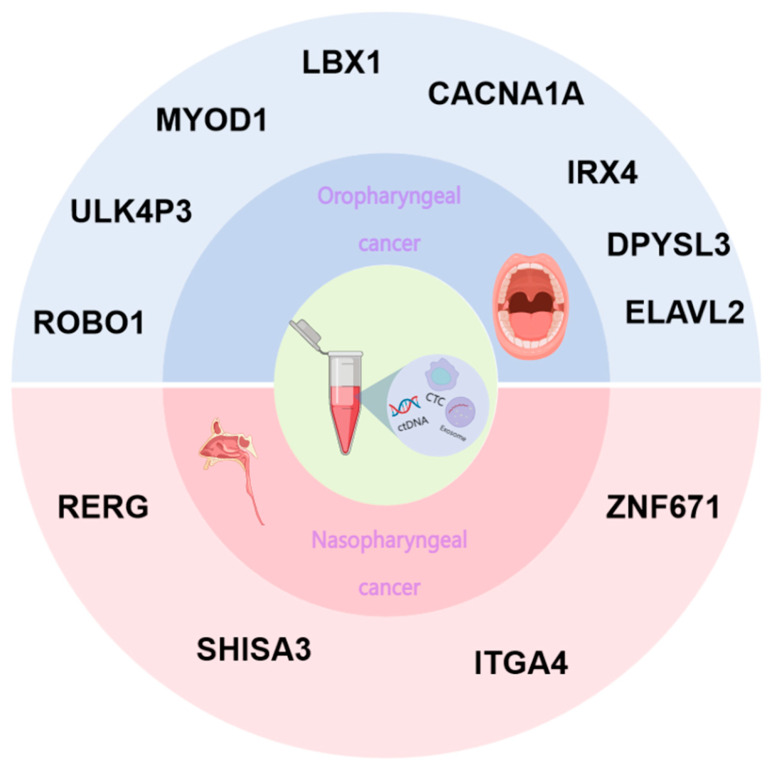
Genes associated with DNA methylation in head tumors currently applied in clinical and research phases.

Nasopharyngeal cancer is a kind of malignant tumor of the head and neck originating from nasopharyngeal epithelial cells. This cancer is common in the south of our country, Southeast Asia, the Middle East, North Africa, etc., and has the characteristics of strong invasiveness and metastasis [38,39]. Nasopharyngeal cancer has a significant ethnic and geographic distribution. Because its early symptoms are not obvious and the mechanism of occurrence and development has not been clarified, early diagnosis is challenging and the statistical analysis of the net survival rate of nasopharyngeal cancer patients in 5 years is only 47% [40,41]. In general, the survival rate and prognosis of patients are poor. Although the therapeutic effect of nasopharyngeal cancer is relatively considerable, recurrence and metastasis remain challenging problems in current treatments. Therefore, the mechanism of nasopharyngeal cancer development needs to be further analyzed, especially in the clinic, and there is an urgent need to find potential early screening biomarkers to improve patient survival. Some studies have revealed that the occurrence and development of nasopharyngeal carcinomas can be influenced by DNA methylation, which regulates gene expression, highlighting the clinical value of DNA methylation detection in the early diagnosis and treatment evaluation of nasopharyngeal carcinomas. Nasopharyngeal cancer is a complex disease linked to specific ethnic groups and family history, and its occurrence involves genetic and environmental factors and their interactions. A meta-analysis of the methylation level of RASSF1A showed that it was significantly higher in early-stage tissue samples of nasopharyngeal carcinoma and in exfoliated cells from nasal mucosa brushes than in healthy individuals [42]. The methylation analysis of the genes EBNA1, LMP1, RASSF1A, DAK, ITGA9, P16, WNT7A, CHFR, CYB5R2, WIF1, RIZ1, and FSTL1 [43] in Moroccan and Southern Chinese patients with NPC revealed differences in DNA methylation sensitivity and specificity among NPC populations from these different regions. Xu [44] quantitatively analyzed DNA methylation, pre-treated tissue DNA, and circulating cell-free DNA (ccfDNA) in blood from 79 nasopharyngeal carcinoma patients and 29 non-cancerous patients using real-time fluorescence quantitative PCR. The results showed that the levels of methylation in the RERG, ZNF671, ITGA4, and SHISA3 genes were notably higher in cancerous tissues compared to non-cancerous tissues. The higher methylation levels of RERG in ccfDNA were also found to be statistically significant, while ZNF671, ITGA4, and SHISA3 did not have significant differences. In addition, the combined detection of methylation levels of RERG and ZNF671 in ccfDNA was found to be have greater diagnostic value than separate detection. It reveals that ccfDNA methylation could be a promising new biomarker for the early detection of nasopharyngeal carcinoma. Meanwhile, a large number of studies in the literature reported that DNA methylation has the ability to forecast clinical outcomes for patients with nasopharyngeal cancer. VIRMA, an m6A writer, is markedly overexpressed in NPC and is crucial for the processes of tumor formation and metastasis in both in vitro and in vivo settings. DNA methylation analysis was performed by dividing patients after nasopharyngeal cancer treatment into two groups with good and poor prognosis, and it was found that hypermethylation of VIRMA was associated with poor outcomes in patients with NPC, which can act as a prognostic biomarker for nasopharyngeal carcinoma [45,46]. Detecting changes in methylation levels of nasopharyngeal cancer-related genes through long-term follow-up can provide important indications of patient prognosis. For nasopharyngeal cancer screening, DNA methylation levels can be measured by detecting circulating cell-free DNA (ccfDNA) in the peripheral blood, saliva, or biopsy tissues of patients using quantitative real-time PCR (qPCR), and the results showed a high degree of consistency, which provides an idea for creating a new method for the non-invasive screening of nasopharyngeal carcinomas [47,48]. The present analysis demonstrates the significant utility of DNA methylation research in the diagnosis and treatment of nasopharyngeal cancer (Figure 2).

### 3.2. DNA Methylation and Thoracic Tumors

Esophageal cancer (EC) ranks among the most prevalent cancers affecting the gastrointestinal system globally. In Western countries, the main subtype is esophageal adenocarcinoma, and esophageal squamous-cell carcinoma (ESCC) is the most common histological type of esophageal cancer. It has the seventh- and sixth-highest incidence and mortality rate in the world [49]. The prevalence and mortality rates related to esophageal cancer in China show obvious gender, age, and regional differences; the incidence and mortality rates of males, those older than 40, and those in rural areas are higher than those of females, those younger than 40, and those in urban areas, respectively [50]. The insidious onset of esophageal cancer often leads to a diagnosis in the middle to late stages, with recurrence and metastasis being the primary contributors to the high mortality rates among patients [51]. However, for early esophageal cancer that only involves the mucosal layer and the superficial submucosal layer, the 5-year survival rate can be as high as 80% after endoscopic resection or minimally invasive surgery [52]. On the other hand, patients diagnosed in the mid-to-late stages usually have a 5-year survival rate of below 20%. Therefore, there is an urgent need to find methods that are applicable to early screening. A substantial body of literature highlights the critical role of DNA methylation, as an epigenetic mechanism, in the pathogenesis of esophageal squamous-cell carcinoma. P16^ink4A^ is a common anti-oncogene inactivated by epigenetic alterations in malignant tumors. Methylation of the P16^ink4A^ promoter leads to the deletion of p16 gene expression during esophageal cancer [53,54]. A study analyzing DNA methylation in Japanese patients with ESCC compared with those from other regions ultimately led to the identification of a gene expression profile for esophageal cancer in Japan, revealing the possible role of AEI and the aberrant expression of imprinted genes in the pathogenesis of esophageal squamous carcinoma [55]. Moreover, DNA methylation exhibits immense potential as a novel biomarker with diverse applications in the diagnostic screening of this malignancy [56]. The fragile histidine triad diadenosine triphosphatase (FHIT) gene was found to be associated with genomic stability and tumor progression, and methylation of the FHIT gene usually occurs during the initial phase of esophageal squamous-cell carcinoma. FHIT gene methylation usually occurs in the early stage of esophageal squamous-cell carcinoma and is significantly correlated with smoking, which can be used as a dormant biomarker for early esophageal squamous-cell carcinoma, greatly improving the diagnostic rate of esophageal squamous-cell carcinoma in the early stage of screening. Studies [57] have shown that the degree of CHFR methylation may be treated as a biomarker in the early stages of esophageal squamous-cell carcinoma. CHFR methylation can be used to determine the extent of esophageal squamous-cell carcinoma and the efficacy of chemotherapy with docetaxel and paclitaxel [58]. The WNT/β-catenin signaling pathway has been found to be intricately associated with the development of human esophageal squamous-cell carcinoma [59]. APC is an inhibitor of the WNT pathway, and it was found that esophageal cancer patients with hypermethylated *APC* had fewer lymph node metastases and better prognosis, which is considered to be a prognostic marker for esophageal cancer [60]. Overall, DNA methylation can be employed as a marker for early screening, diagnosis and treatment, prognosis assessment, and chemotherapy sensitivity in esophageal squamous-cell carcinoma.

Lung cancer is currently the leading malignant tumor in the world in terms of mortality and morbidity, and smoking is the main factor leading to the development of lung cancer. The vast majority of lung cancer patients are typically diagnosed at advanced stages, resulting in a significantly unfavorable prognosis. For the diagnosis of lung cancer, pathological diagnosis is the gold standard [61]. Currently, the implementation of low-dose computed tomography scanning for lung cancer has the potential to significantly enhance the screening rate among patients; however, it is associated with a considerable rate of false positives. So far, screening, diagnostic, and therapeutic methods for lung cancer still face great challenges. Hypermethylation of local regions was found in patients with malignant tumors. On the other hand, regions such as anti-oncogene promoter regions showed hypomethylation at the genomic level [62]. RASSF1A, as an important anti-oncogene, is involved in tumorigenesis through the Ras signaling pathway. Hypermethylation of the RASSF1A promoter mediates DNMT3b expression through HOXB3 [63]. Therefore, researchers believe that DNA methylation has the potential to be a novel biomarker for lung cancer. DNA methylation has been reported in lung cancer. Studies have shown that SOX17, RASSF1A, and SHOX2 methylation can be applied as markers for lung cancer screening and detection [64,65]. Researchers further found that the combination of multiple genes is more sensitive than a single gene in diagnosing lung cancer [66]. Currently, domestic and international studies have found that abnormal methylation is found in peripheral blood, sputum, and alveolar lavage fluid. Hulbert [67] found that the combination of three genes, SOX17, TAC1, and HOXA7, could differentiate between individuals with lung cancer and those with benign nodules, and the sensitivity of their individual genes was only about 70%, while the sensitivity of the three genes for the combined detection of lung cancer reached 93% and 98% in blood and sputum, respectively. The DNA methylation levels of cancer tissues, blood, and sputum in lung cancer patients showed a high degree of consistency, suggesting that the testing of DNA methylation holds great significance in the non-invasive screening and diagnosis of lung cancer. Analysis of cancerous and normal tissues from lung cancer patients using 450 K microarrays reveals that the larger the tumor (>3 cm), the older the age of the patient (>65 years), and the higher the recurrence rate is after surgery, the higher the methylation level [68]. Numerous investigations have demonstrated that DNA methylation assumes a vital role in cisplatin resistance in lung cancer. hMLH1 promoter methylation [69] can lead to the occurrence of DDP resistance in vitro and in vivo, which can affect the therapeutic effect of patients, revealing that hMLH1 is capable of functioning as a biomarker for the individualized treatment of lung cancer. The biomarker hMLH1 can be utilized to personalize the treatment of lung cancer. Nehme [70] pointed out that the methylation status of the T-BOX transcription factor 2 subfamily can be used as an indicator of the clinical response of NSCLC to the anti-tumor drug 5-azacytidine, suggesting that DNA methylation testing also plays a great role in monitoring the disease progression of lung cancer patients. Studies have shown that the degree of DNA methylation is significantly different in different stages of lung cancer, and in NSCLC, the degree of DNA methylation can reflect the progression of the tumor [71,72,73]. Meanwhile, certain studies have identified specific CpGs that demonstrate age-related alterations in diverse tissues, with the age of DNA methylation significantly associated with biological age. In patients with lung adenocarcinoma, there was a notable disparity between the biological age of the tumor and the actual age of the patient [74].

**Figure 3:**
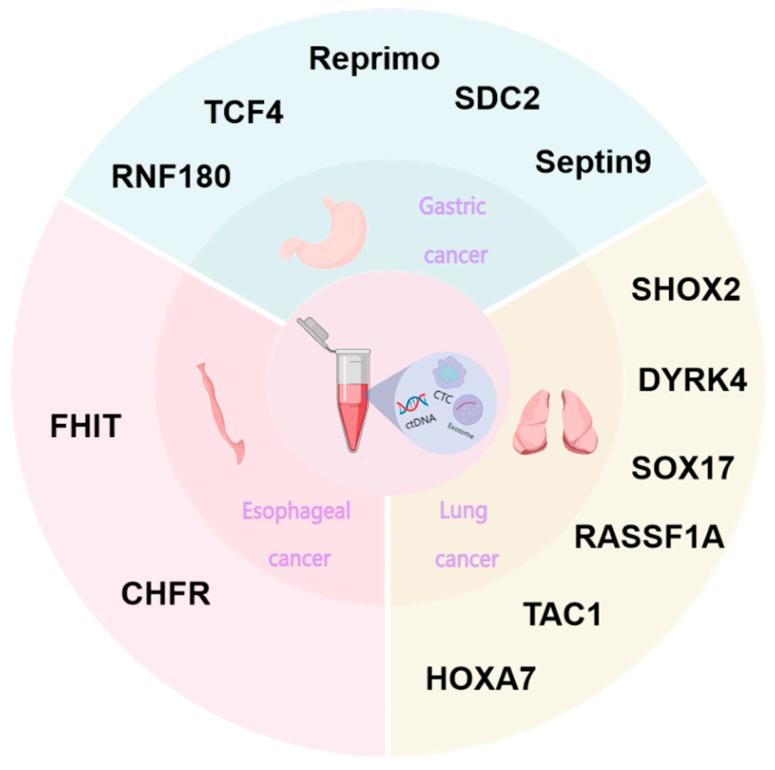
Genes associated with DNA methylation in thoracic tumors currently applied in clinical and research phases.

Gastric cancer is the malignant neoplasm of the gastrointestinal system with the highest incidence rate in China, ranking third in terms of mortality rate. The statistics indicate that the median overall survival time of gastric cancer patients is merely 16 months [75]. The majority of patients are initially diagnosed at an intermediate to advanced stage, which is associated with a dismal treatment prognosis and limited survival duration. In order to improve the 5-year survival rate of patients, early detection and treatment are undoubtedly the key to solving the problem. However, the clinical symptoms of early gastric cancer are insidious, and the sensitivity of imaging examinations for the diagnosis of early gastric cancer is poor, which brings certain difficulties to early diagnosis. The latest research indicates that the methylation status of DNA has been changed in precancerous lesions, which can be utilized for non-invasive early screening and diagnosis of gastric cancer. The 2023 Chinese Expert Consensus on Early Gastric Cancer Screening and Testing Techniques states that RS19 gene methylation, combined with RNF180 and Septin9, can be used for the detection of early gastric cancer, suggesting that positive subjects should undergo gastroscopy in a timely manner. The incidence of gastric cancer increases with age and a common sign of aging and cancer is changes in DNA methylation patterns. Some studies have shown CGI hypermethylation and overall DNA hypomethylation in the promoter region of centenarians, which is similar to the DNA methylation pattern in cancer. For example, the promoter regions of ERα and p16INK4a, which are anti-oncogenes, are susceptible to hypermethylation in senescent cells [76]. In recent years, the use of serum tumor marker tests in clinical practice has become increasingly widespread, and the detection of relevant serum tumor markers such as CEA, CA125, and CA199 can be used to screen some malignant tumors [77]. In addition, the combination of DNA methylation and tumor markers can further enhance the diagnostic sensitivity of gastric cancer. In addition, many studies have found that aberrant gene methylation modifications are linked to recurrence, metastasis, and response to treatment [78]. Chen [79] found that gastric cancers with a higher degree of CpG island methylation had more distant lymph node metastases and pathologic differentiation through the detection of the methylation status of ALX2, TMEFF10, CHCHD3, IGFBP1, and NPR0. The metastasis was more distant and the type of pathological differentiation was worse. The suggestion arises that the modification of DNA methylation status confers a notable advantage in monitoring the prognosis of patients. Therefore, DNA methylation can serve as a tool for the early diagnosis of gastric cancer, monitoring the progression and recurrence of the tumor during treatment, and predicting prognoses (Figure 3).

### 3.3. DNA Methylation and Abdominal Tumors

Liver cancer is one of the most common forms of cancer worldwide. Hepatocellular carcinoma is the main histological type of hepatocellular carcinoma, accounting for 85% to 90% of liver cancers. Liver cancer in China is mainly dominated by HBV infection, accounting for about 86% of cases, along with long-term alcohol consumption and long-term use of aflatoxin-contaminated food [80]. The early diagnosis of hepatocellular carcinoma plays a crucial role in enhancing the prognosis. Currently, ultrasound and alpha-fetoprotein (AFP) are the preferred monitoring tools for the recurrence of hepatocellular carcinoma, but because of the low specificity, there is still a need to seek more accurate biomarkers to monitor the recurrence of hepatocellular carcinoma and to improve patient survival. Research has demonstrated that DNA methylation has potential advantages in diagnosing and monitoring the prognosis of hepatocellular carcinoma [81,82]. A meta-analysis [83] revealed that dysregulated DNA methylation may be a useful biomarker for the prediction and diagnosis of liver cancer, and the timely diagnosis of hepatocellular carcinoma plays a crucial role in improving the prognosis. A study [84] evaluated the efficacy of SEPT9 promoter methylation (mSEPT9) in the plasma of 104 patients with hepatocellular carcinoma and 174 healthy people for identifying hepatocellular carcinoma. The results showed that in the hepatocellular carcinoma group and the healthy control group, the sensitivity of mSEPT9 promoter methylation was 82.7%, the specificity was 96.0%, and the AUROC was 0.961 (95% CI: 0.933). The sensitivity of mSEPT9 promoter methylation was 82.7%, while the specificity was 96.0%. Moreover, the AUROC was 0.961 (95% CI: 0.933–0.989), which demonstrated a significantly superior performance compared to AFP (AUROC was 0.881, sensitivity was 57.7%, and specificity was 98.3%). This revealed that mSEPT9 is potentially valuable for the early diagnosis of hepatocellular carcinoma. Some studies have shown that by detecting the changes in plasma methylation levels of BMPR1A and PLAC8 genes in patients with and without recurrence of hepatocellular carcinoma and comparing them with the traditional tumor marker AFP in predicting the recurrence of hepatocellular carcinoma, the results indicated that the sensitivity and specificity of the combined detection of BMPR1A and PLAC8 methylation in predicting the recurrence of hepatocellular carcinoma were 66.7% and 88.9%. The detection of BMPR1A was expected to be more sensitive and specific than the detection of PLAC8 gene methylation in patients with hepatocellular carcinoma recurrence, and PLAC8 methylation is expected to be a method that can be used to monitor the recurrence of hepatocellular carcinoma.

Colorectal cancer, ranked as the third most prevalent malignancy globally, is also among the major contributors to cancer-related mortality in the world. Currently, the incidence or death rate of colorectal cancer is still showing an increasing trend year by year. The prevalence of colorectal cancer is related to a variety of factors such as dietary habits, heredity, colitis, etc. [85]. It is usually the result of the joint action of genes and the environment. In recent years, the incidence of colorectal malignancies in developed Western nations has been on a downward trend, especially in the age group of 50–74 years, which is intricately related to the prevention and treatment of precancerous lesions. Various studies have consistently demonstrated that the cure rate of colorectal cancer can reach more than 90% when detected at an early stage. On the other hand, it is less than 10% when detected at an advanced stage. Therefore, early diagnosis and treatment are key to improving the prognosis of colorectal cancer. Currently, the gold standard for colorectal cancer screening is still colonoscopy, but its invasive and cumbersome nature affects patient compliance [86]. A number of studies have demonstrated the potential of DNA methylation markers in the early detection of colorectal cancer [87,88], with aberrant methylation markers detected in tumor tissues, feces [89], and blood [90]. As the first and only FDA-approved methylation biomarker, SEPT9 has been widely studied by researchers. Warren [91] found that plasma SEPT9 had a sensitivity and specificity of 90% and 88%, respectively, for the diagnosis of colorectal cancer in a retrospective case-control analysis. A study [92] suggests that SEPT9 in feces has more diagnostic value for colorectal cancer than plasma SEPT9, but a large number of clinical trials are required to validate this. The researchers suggested that the simultaneous evaluation of SFRP1, SFRP2, SDC2, and PRIMA1 methylation has great diagnostic value for colorectal cancer by studying the combined detection of multiple genes [93]. Currently, single-gene markers show good diagnostic value in the early screening of colorectal cancer, but their sensitivity is not stable. Multi-gene combined detection has received widespread attention and research, but further studies are needed to verify its diagnostic value.

Bladder cancer is the malignant tumor of the urinary system with the highest incidence rate, and the incidence rate in China has been gradually increasing in recent years. According to the “Analysis of the prevalence of malignant tumors in China” published in 2024, bladder cancer ranked eighth in the incidence of malignant tumors among men in China, with an incidence of 73,200 cases. Studies have shown that most bladder cancers are prone to recurrence and progression from non-muscle invasive carcinoma to muscle invasive carcinoma, with poor prognosis. Currently, diagnosis and postoperative follow-up still rely on invasive cystoscopy, which tends to cause discomfort to patients and lacks effective treatment for progressive tumors. However, the main test for bladder cancer is urine exfoliative cytology, but its sensitivity is low. Therefore, there is a need to find new, highly sensitive, and specific tumor molecular biomarkers to provide new options for the early diagnosis, treatment, and prognosis of bladder cancer. Sanford [94] identified 240 differentially methylated genes through the analysis of methylation and transcriptional data of bladder cancer using the combined Cancer Genome Atlas (TCGA), suggesting the presence of altered DNA methylation levels in bladder cancer. Wu [95] established a combination of methylation markers for four genes (HOXA9, procalcitonin 17, POU4 homology cassette gene 2, and one cut homology cassette gene 2) through the methylation analysis of urine samples from 192 patients with hematuria, and the results showed a sensitivity of 73% and a specificity of 91%, suggesting that DNA methylation testing can be used for the early detection of bladder cancer. This finding implies that DNA methylation testing could represent a valuable method for the early detection of bladder cancer. Van Kessel [96] detected the presence of DNA in the urine of patients with hematuria, and found that the methylation levels of combined TWIST1, ONECUT2, and Orthodenticle human homology cassette gene 1 had a high sensitivity (97%) and specificity (83%). On this basis, the combination of FGFR3, TERT, and HRAS proto-oncogene mutations resulted in a sensitivity of 93% and a specificity of 89% for the diagnosis of bladder cancer, which shows that the combination of DNA methylation testing has great potential in the detection of bladder cancer. Stubendorff [97] conducted a genome-wide methylation analysis of bladder cancer in 23 bladder cancer patients and found that the DNA methylation levels of the two genomes had high sensitivity (97%) and specificity (83%) for the diagnosis of bladder cancer in patients with hematuria. A comprehensive genome-wide methylation analysis was carried out and later confirmed, and a combination of markers containing the methylation of three genes (kisspeptin receptor gene, SEPTIN9, and cysteine sulfinic acid decarboxylase gene) was identified. The combined methylation level of the markers can be utilized for the prediction of post-surgical metastasis risk in bladder cancer patients. Thus, it can be seen that DNA methylation detection has great application prospects in the early diagnosis and prognosis monitoring of bladder cancer.

**Figure 4:**
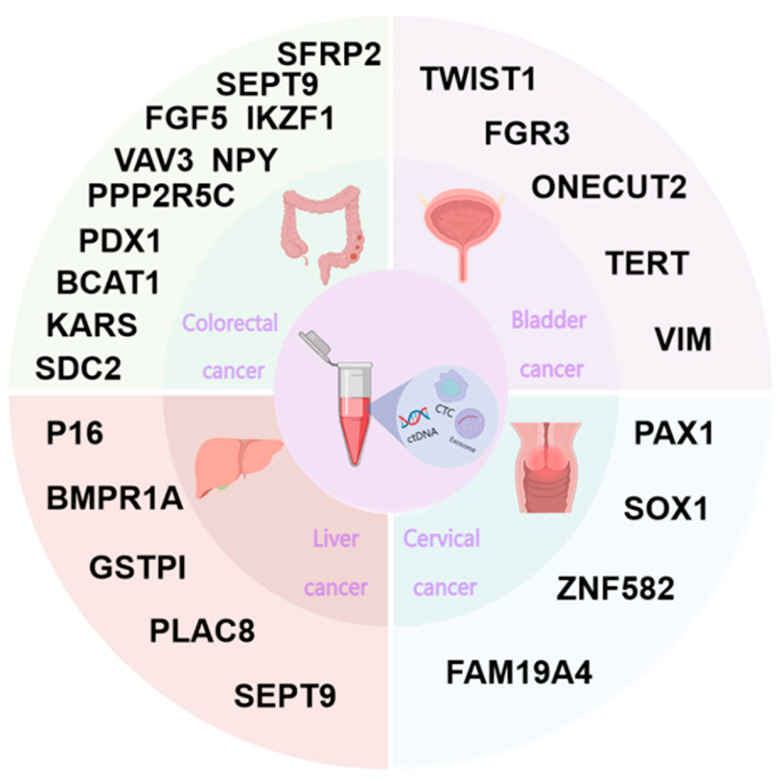
Genes associated with DNA methylation in abdominal tumors currently applied in clinical and research phases.

Cervical cancer is the most common malignant tumor among women in the world. The incidence of cervical cancer in China is increasing year by year, and it is the country with the second-largest cervical cancer disease burden in the world. Human papillomavirus (HPV) infection is the predominant etiological factor associated with the onset of cervical cancer, associated with more than 99% of cervical squamous-cell carcinoma cases worldwide [98]. Currently, high-risk HPV testing and cytology are the routine cervical cancer screening methods in China, but there is a risk of misdiagnosis or omission of cervical cancer due to low specificity or sensitivity. The overall survival rate at five years for patients diagnosed with cervical cancer has been reported to be 68%, while the survival rate of patients with recurrent or metastatic cervical cancer is still poor at only 17% [99]. Therefore, a significant demand exists for reliable approaches to the early diagnosis and management of cervical cancer. Studies have shown that DNA methylation is closely associated with the development of cervical cancer and precancerous lesions, and that methylation levels of specific host cell gene promoter regions increase with the development of cervical intraepithelial neoplasia (CIN) lesions. A study of methylation analysis of the DPP6, RALYL and GSX1 gene regions in a cohort of women aged ≥30 years attending cervical cancer screening in the Stockholm region of Sweden revealed that early screening for cervical cancer can be effectively performed by detecting the degree of methylation of the three genes in conjunction with HPV16/18 genotyping. However, this study has not been validated in other regional populations [100]. The risk of cervical carcinogenesis was analyzed by simultaneous detection of methylation levels of human ASTN1, DLX1, ITGA4, RXFP3, SOX17, and ZNF671 genes by fluorescent PCR [101]. The incidence of methylation was found to be 100%, 88%, 83%, and 17% in cervical cancer, high-grade lesions, low-grade lesions, and normal controls, respectively, which verified the significant correlation between DNA methylation and cervical intraepithelial neoplasia. Numerous findings have shown that PAX1, SOX1, and ZNF582 methylation tests are more accurate for the diagnosis of cervical cancer compared to exfoliative cytology [102]. The sensitivity of FAM19A4/miR124-2 methylation in HPV-positive CIN3 and cervical cancer was determined to be 95% and 77.2% respectively, and the specificity was found to be 78.3%, which suggests that DNA methylation can be applied to the early screening of cervical cancer [103]. Meanwhile, some studies have also shown that methylation testing can be used to predict the survival rate of patients. In conclusion, the utilization of DNA methylation shows promise for the precise screening of cervical cancer and has the potential to predict outcomes in patients with this disease (Figure 4).

## 4. Summary

Cancer has consistently been a leading contributor to the ongoing rise in global mortality rates, and the occurrence of tumors is a multifactorial event. DNA methylation, as one of the most important modifications of epigenetics, plays an important role in the development of tumors. Liquid biopsy has been widely noticed by clinics because of its convenient, easy, and repeatable sampling, etc. Many scholars are actively exploring the potential of DNA methylation-based liquid biopsy in early cancer screening and prognosis monitoring. Currently, the most important technology for methylation detection is still bisulfite combined with sequencing, and improving the existing technology to obtain a sensitive, specific, easy-to-operate, and high-throughput detection method is still the focus of future research. Existing reports have shown that DNA methylation has great potential in cancer diagnosis and screening! DNA methylation exerts a significant impact on the incidence, mortality, therapeutic response, and prognosis of diverse malignancies. Moreover, DNA methylation levels are also associated with tumor patients from distinct geographical regions, age groups, and genders. The methylation of the SEPT8 gene was found to be significantly different between Xinjiang Uyghurs and Han Chinese populations. The methylation of EBNA1, LMP1, R ASSF1A, DAK, ITGA9, P16, WNT7A, CHFR, CYB5R2, WIF1, RIZ1, and FSTL1 genes was found to be significantly different in Moroccan patients with NPC compared to patients with NPC in Southern China. The aberrant expression of methylation in AEI and imprinted genes in Japanese patients with esophageal cancer was also found. Detecting the methylation of the DPP6, RALYL, and GSX1 genes was also used for the early diagnosis of cervical cancer in women in Stockholm, Sweden. However, limited investigations have been conducted in this domain, and DNA methylation differences in other clinical tumors in different populations are currently less well studied. And there are still some limitations. The mechanism of abnormal methylation leading to tumorigenesis has not been fully explored, and the biggest challenge facing methylation marker research remains the discovery and validation of early cancer markers. However, due to the large number of types of cancers and the relatively small number of cases, etc., more research is needed before DNA methylation-based liquid biopsy can be truly applied to the clinic in its entirety. We are optimistic about the prospects of DNA methylation detection technology in clinical settings, as it has the potential to significantly improve early cancer diagnosis, prognosis assessment, and comprehensive clinical support.

## Data Availability

Not applicable.
